# Handling missing data when estimating causal effects with targeted maximum likelihood estimation

**DOI:** 10.1093/aje/kwae012

**Published:** 2024-02-22

**Authors:** S Ghazaleh Dashti, Katherine J Lee, Julie A Simpson, Ian R White, John B Carlin, Margarita Moreno-Betancur

**Keywords:** missing data, causal inference, targeted maximum likelihood estimation, multiple imputation

## Abstract

Targeted maximum likelihood estimation (TMLE) is increasingly used for doubly robust causal inference, but how missing data should be handled when using TMLE with data-adaptive approaches is unclear. Based on data (1992-1998) from the Victorian Adolescent Health Cohort Study, we conducted a simulation study to evaluate 8 missing-data methods in this context: complete-case analysis, extended TMLE incorporating an outcome-missingness model, the missing covariate missing indicator method, and 5 multiple imputation (MI) approaches using parametric or machine-learning models. We considered 6 scenarios that varied in terms of exposure/outcome generation models (presence of confounder-confounder interactions) and missingness mechanisms (whether outcome influenced missingness in other variables and presence of interaction/nonlinear terms in missingness models). Complete-case analysis and extended TMLE had small biases when outcome did not influence missingness in other variables. Parametric MI without interactions had large bias when exposure/outcome generation models included interactions. Parametric MI including interactions performed best in bias and variance reduction across all settings, except when missingness models included a nonlinear term. When choosing a method for handling missing data in the context of TMLE, researchers must consider the missingness mechanism and, for MI, compatibility with the analysis method. In many settings, a parametric MI approach that incorporates interactions and nonlinearities is expected to perform well.

## Introduction

A key component of epidemiologic research is causal inference from longitudinal studies, where the objective is often to estimate the average causal effect (ACE) of an exposure on an outcome.^1^^-^^5^ For a binary exposure ($X$=1 exposed; $X$=0 unexposed), the ACE can be defined as the difference in the average potential outcome if all participants were exposed versus unexposed.^1^^‑^^5^ In the absence of missing data, under the assumptions of conditional exchangeability given a vector of measured confounders $\boldsymbol{Z}$, consistency, and positivity, the ACE is identifiable from observable data by the g-formula $\mathrm{E}\left[\mathrm{E}\left(Y|X=1,\boldsymbol{Z}\right)-\mathrm{E}\left(Y|X=0,\boldsymbol{Z}\right)\right]$, where $Y$ is the outcome.^6^

Several singly robust approaches, including g-computation and propensity score methods, and doubly robust estimators, including targeted maximum likelihood estimation (TMLE), are available for ACE estimation in the absence of missing data. Here, we focus on TMLE, which combines models for the outcome and propensity score.^7^^‑^^9^ A detailed description of TMLE is available elsewhere.^7^^‑^^9^ Briefly, the first step is the same as in g-computation, where a model for the expected outcome conditional on exposure and confounders ($\hat{\mathrm{E}}\left[Y|X,\boldsymbol{Z}\right]$) is fitted and used to predict outcomes for all records under exposure and no exposure. In g-computation these predictions are directly plugged into the g-formula to estimate the ACE. In TMLE, they are updated using information from the propensity score ($\hat{P}\left[X=1|\boldsymbol{Z}\right]$) (the targeting step) before being plugged into the g-formula.^7^ The targeting step in TMLE ensures that the estimator is doubly robust, where only 1 of the 2 models (outcome or propensity model) needs to be consistently estimated to ensure consistent estimation of the ACE. Unlike with singly robust methods, data-adaptive approaches (eg, machine learning methods) can be used for the exposure and outcome models in TMLE as long as the Donsker class condition (requiring that outcome and propensity score estimators do not heavily overfit the data) holds.^8^^‑^^10^ Given these desirable properties, interest in the application of TMLE for ACE estimation is growing.

Missing data are ubiquitous in epidemiologic studies and can lead to biased estimates and loss of precision if handled inappropriately.^11^ Commonly used approaches for handling missing data in studies using TMLE for ACE estimation include multiple imputation (MI; eg, see Yu et al^12^), complete-case analysis (CCA; eg, see Bell-Gorrod et al^13^), extension of TMLE to handle missing outcome data (eg, see Rossides et al^14^), and the missing covariate missing indicator (MCMI) approach for handling missing confounder data (eg, see Ehrlich et al^15^). To the best of our knowledge, no study has compared these methods in terms of bias and precision. In addition, while a requirement for valid inference with MI is that the imputation model should incorporate all relationships assumed to hold in the analysis method,^11^ the optimal implementation of MI when using TMLE with data-adaptive methods for ACE estimation is unknown. Answers to these questions are key to developing guidance for appropriate handling of missing data in the context of the growing use of TMLE in applied epidemiologic research.

In the current paper, we seek to address this knowledge gap using a simulation study based on an illustrative example from the Victorian Adolescent Health Cohort Study (VAHCS). Our interest is to compare the performance of readily available missing-data methods to inform current practice. We begin by introducing the VAHCS example and then describe methods for handling missing data with TMLE, present results from the simulation study we conducted to evaluate and compare the performance of these approaches, and illustrate the assessed approaches in the VAHCS example. We conclude with a general discussion.

## Methods

### Illustrative example

Our example was based on a previous investigation using data from VAHCS, a longitudinal cohort study of 1943 participants (1000 females) recruited in 1992-1993 at ages 14-15 years.^16^ Data were collected during participants’ adolescence (waves 1-6) and in young adulthood (1998, wave 7). Investigators aimed to estimate the ACE of frequent cannabis use in adolescent females on mental health in young adulthood, measured using the Clinical Interview Schedule–Revised.^17^ We revisited this analysis using TMLE with data-adaptive approaches, using the same confounders as those considered in the original investigation: parental divorce, antisocial behavior, depression and anxiety, alcohol use, and parental education, all measured across waves 2-6.^18^  Table 1 shows descriptive statistics for analysis variables, as well as age at wave 2, which is useful as an auxiliary variable in MI (a predictor of missing values but not included in the analysis method^11^). All variables had some degree of missingness, ranging from 0.1% to 30.8%.

**Table 1:** Variables used in the case study, their distributions, and proportions with missing data among female participants (*n* = 1000), Victorian Adolescent Health Cohort Study, 1992-1998.

**Component**	**Variable**	**Variable type**	**Grouping/unit**	**Notation**	**No. (**%^**a**^**) coded 1 or ** **mean (SD)**	**% with** **missing data**
Confounder	Parental divorce	Binary	0 = Not divorced/separated by wave 6 1 = Divorced/separated by wave 6	$Z$ _1_	221 (22.1)	0.1
	Antisocial behavior	Binary	0 = No across all waves 2-6 1 = Yes at any wave 2-6	$Z$ _2_	106 (14.6)	27.4
	Depression and anxiety	Binary	0 = CIS-R score <12 across all waves 2-6 1 = CIS-R score ≥12 at any wave 2-6	$Z$ _3_	516 (59.9)	13.8
	Alcohol use	Binary	0 = No across all waves 2-6 1 = Yes at any wave 2-6	$Z$ _4_	294 (37.2)	21.0
	Parental education	Binary	0 = Did not complete high school by wave 6 1 = Completed high school by wave 6	$Z$ _5_	364 (37.7)	3.4
Exposure	Frequent cannabis use	Binary	0 = Less than weekly use across all waves 2-6 1 = At least weekly use at any wave 2-6	$X$	86 (12.4)	30.8
Outcome	CIS-R total score	Continuous	*z* score, measured at wave 7	$Y$	0 (1)	13.4
Auxiliary variable	Age	Continuous	Years, measured at wave 2	$A$	15.4 (0.4)	9.3
With any missing data						40.3

Abbreviation: CIS-R, Clinical Interview Schedule–Revised.

^a^ Proportions are reported among persons with observed data for the variable.

### Methods for handling missing data in TMLE

Approaches proposed for handling missing data when estimating the ACE using TMLE in studies like our example are described below.

#### Non-MI approaches

##### Complete-case analysis

In CCA, only records with complete data for target analysis variables are used.^19^ This approach generally leads to loss of precision^20^ and, depending on the missingness mechanism, may inflict bias.^19^

##### Extended TMLE in a sample with complete exposure and confounders

In extended TMLE in a sample with complete exposure and confounders (Ext-TMLE+CEC), records with missing data for $\boldsymbol{Z}$ and $X$ are deleted. From records with complete data on $Y$, $\mathrm{E}\left[Y|X,\boldsymbol{Z}\right]$ is estimated. In the targeting step, the predictions of the outcome are updated using information from models for *P*[*X* = 1|***Z***] and *P*[${M}_Y$=0|*X*, ***Z***], where ${M}_Y$ is the missingness indicator for the outcome.^21^ Updated predictions for the outcome under exposure and no exposure are obtained for all records, regardless of whether they have missing outcome.^21^ The model for ${M}_Y$ can also be fitted using data-adaptive approaches. With missingness only in the outcome, the extended TMLE method is unbiased under an extended exchangeability assumption (${Y}^x\coprod{M}_Y\mid X,\boldsymbol{Z}$ and ${Y}^x\coprod X\mid \boldsymbol{Z}$ for $x=0,1,$ where ${Y}^x$ is the potential outcome when $X=x$, and ∐ denotes independence).^22^

##### Extended TMLE plus MCMI

In the extended TMLE plus MCMI (Ext-TMLE+MCMI) approach, missing outcome data are handled using the extended TMLE approach, and missing confounder data by including missingness indicators for the incomplete confounders in the confounding adjustment set. Records with missing exposure data are excluded. In settings with complete exposure and outcome data, the MCMI approach can be expected to yield an unbiased ACE estimate under an extended exchangeability assumption (${Y}^x\coprod X\mid \boldsymbol{Z},{\boldsymbol{M}}_{\boldsymbol{Z}}$ for $x=0,1,$ where ${\boldsymbol{M}}_{\boldsymbol{Z}}$ is the vector of missingness indicators for the incomplete confounders), and the assumption that the exposure or outcome depends on the confounder only when the confounder is observed.^23^^,^^24^ This assumption is plausible in some settings, such as when using electronic health record data, where the decision to prescribe a medication might be influenced by family history of disease only when the clinician has the relevant information.

#### MI approaches

The MI fully conditional specification (FCS) framework^25^ enables simultaneous handling of missing exposure, confounder, and outcome data. Under this approach, univariate imputation models are specified for each incomplete variable conditional on other variables, and imputations are drawn sequentially until convergence.^25^ The process is repeated multiple times to generate multiple completed data sets. Analysis is performed within each completed data set, and the results are pooled using Rubin’s rules to obtain the final estimate and its SE.^25^ For valid inference with MI, each univariate imputation model should be tailored to be compatible with the analysis method. To achieve this, all analysis variables and complexities such as interaction terms in the target analysis should be included as predictors in each univariate imputation model.^25^ There are various possible implementations of MI within the FCS framework.

##### Parametric MI with no interaction

In parametric MI with no interaction (MI-no int), each univariate imputation model is based on a regression model with main-effects terms only—the default model in most MI software. In the example and simulations, we considered main-effects logistic regression for the binary variables and predictive mean matching (PMM) for the continuous outcome based on a main-effects linear regression. In PMM, imputed values are drawn using the nearest observed value after fitting the regression,^25^ which makes it robust to misspecification of the latter—for example, in the presence of nonlinear associations.^26^

##### Parametric MI with 2-way interactions

In parametric MI with 2-way interactions (MI-2-way int), each univariate imputation model is based on a regression model as above, but 2-way exposure-outcome, exposure-confounder, confounder-outcome, and confounder-confounder interactions are included. Interaction terms are generated within each cycle of the MI algorithm from current values of relevant variables involved in the interaction term (the so-called “passive” approach in *mice* in R).^27^

##### Parametric MI with 2-, 3-, and 4-way interactions

Parametric MI with 2-, 3-, and 4-way interactions (MI-higher int) is conducted as above, but all univariate imputation models additionally include 3- and 4-way confounder-confounder interactions as predictors.

##### MI using classification and regression trees

In MI using classification and regression trees (MI-CART), instead of regression, for each variable with missing data a tree is fitted using a recursive partitioning technique, with all other variables as predictors. Each record belongs to a donor leaf, from which a randomly selected value for the variable is taken as the imputed value.^28^ MI-CART (and MI using random forest; see below) have been proposed to enable imputation that can more flexibly allow for interactions and nonlinearities.^28^

##### MI using random forest

In MI using random forest (MI-RF), for each variable with missing data, multiple bootstrap samples are drawn, and for each a separate tree is fitted. Each tree contributes a donor leaf, and a randomly selected value for the variable from all of these donors is taken as the imputed value.^28^

All MI approaches can be implemented with the *mice* package in R.^27^

### Simulation study

**Figure 1:**
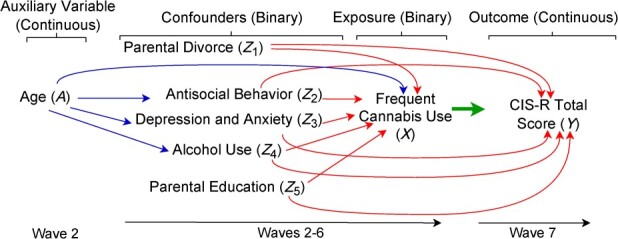
Directed acyclic graph used in data generation for the simulation study, Victorian Adolescent Health Cohort Study, 1992-1998. CIS-R, Clinical Interview Schedule–Revised.

To compare the performance of the described methods for handling missing data, we conducted a simulation study based on the VAHCS example (Figure 1). We considered 6 scenarios. For each scenario, we generated 2000 data sets of 2000 records.

#### Generating the complete data

We used parametric regression models to generate the variables. The values of parameters in the models were determined by fitting similar models to the available data in VAHCS (unless stated otherwise). We considered 2 data-generating scenarios—simple and complex—differing in the confounder-confounder interaction terms used in the data-generation models. No exposure-confounder interaction terms were included (no effect modification). Table S1 provides the parameter values used for simulating the data, and Table S2 gives descriptive statistics for the variables in the simulated data.

For both scenarios, we generated a continuous auxiliary variable $A$ (age at wave 2) and a set of confounders $\boldsymbol{Z}=({Z}_1$ (parental divorce), ${Z}_2$ (antisocial behavior), ${Z}_3$ (depression and anxiety), ${Z}_4$ (alcohol use), ${Z}_5$ (parental education)). The models for generating these variables (all binary variables coded 0/1 and ${\mathrm{logit}}^{-1}\left(\cdot \right)=\exp \left(\cdot \right)/\left[1+\exp \left(\cdot \right)\right]$) were:

$$ A\sim \mathrm{N}\left(0,1\right) $$

$$ {Z}_1\sim \mathrm{Binomial}\left(1,{\mathrm{logit}}^{-1}\left({\mathrm{\alpha}}_0\right)\right) $$

$$ {Z}_2\sim \mathrm{Binomial}\left(1,{\mathrm{logit}}^{-1}\left({\mathrm{\beta}}_0+{\mathrm{\beta}}_1A\right)\right) $$

$$ {Z}_3\sim \mathrm{Binomial}\left(1,{\mathrm{logit}}^{-1}\left({\mathrm{\gamma}}_0+{\mathrm{\gamma}}_1A\right)\right) $$

$$ {Z}_4\sim \mathrm{Binomial}\left(1,{\mathrm{logit}}^{-1}\left({\mathrm{\delta}}_0+{\mathrm{\delta}}_1A\right)\right) $$

$$ {Z}_5\sim \mathrm{Binomial}\left(1,{\mathrm{logit}}^{-1}\left({\mathrm{\zeta}}_0\right)\right) $$

The scenarios differed in the exposure and outcome generation models.

##### Simple scenario

In the simple scenario, we used main-effects regression models to generate a binary exposure $X$ (frequent cannabis use) and a continuous outcome $Y$ (log-transformed, standardized Clinical Interview Schedule–Revised total score):

\begin{align*} {X}_{\mathrm{simple}}\sim \mathrm{Binomial}\Big(1,{\mathrm{logit}}^{-1}\big({\mathrm{\eta}}_0&+{\mathrm{\eta}}_1{Z}_1+{\mathrm{\eta}}_2{Z}_2+{\mathrm{\eta}}_3{Z}_3+{\mathrm{\eta}}_4{Z}_4\\&+{\mathrm{\eta}}_5{Z}_5+{\mathrm{\eta}}_6A\big)\Big) \end{align*}

$$ {Y}_{\mathrm{simple}}\sim \mathrm{N}\left({\mathrm{\theta}}_0+{\mathrm{\theta}}_1X+{\mathrm{\theta}}_2{Z}_1+{\mathrm{\theta}}_3{Z}_2+{\mathrm{\theta}}_4{Z}_3+{\mathrm{\theta}}_5{Z}_4+{\mathrm{\theta}}_6{Z}_5,\mathrm{SD}=1\right) $$

##### Complex scenario

In the complex scenario, we used regression models that included confounder-confounder interactions (excluding interactions with ${Z}_2$ because of the low prevalence (15%)) to generate the exposure and outcome:

\begin{align*} {X}_{\mathrm{complex}}&\sim \mathrm{Binomial}\Big(1,{\mathrm{logit}}^{-1}\big({\mathrm{\eta}}_0^{\ast }+{\mathrm{\eta}}_1{Z}_1+{\mathrm{\eta}}_2{Z}_2+{\mathrm{\eta}}_3{Z}_3+{\mathrm{\eta}}_4{Z}_4\\&\quad+{\mathrm{\eta}}_5{Z}_5+{\mathrm{\eta}}_6A+{\mathrm{\eta}}_7{Z}_1{Z}_3+{\mathrm{\eta}}_8{Z}_1{Z}_4+{\mathrm{\eta}}_9{Z}_1{Z}_5+{\mathrm{\eta}}_{10}{Z}_3{Z}_4\\&\quad+{\mathrm{\eta}}_{11}{Z}_3{Z}_5+{\mathrm{\eta}}_{12}{Z}_4{Z}_5\big)\Big) \end{align*}

\begin{align*} {Y}_{\mathrm{complex}}&\sim N\big({\mathrm{\theta}}_0^{\ast }+{\mathrm{\theta}}_1X+{\mathrm{\theta}}_2{Z}_1+{\mathrm{\theta}}_3{Z}_2+{\mathrm{\theta}}_4{Z}_3+{\mathrm{\theta}}_5{Z}_4+{\mathrm{\theta}}_6{Z}_5+{\mathrm{\theta}}_7{Z}_1{Z}_3\\&\quad+{\mathrm{\theta}}_8{Z}_1{Z}_4+{\mathrm{\theta}}_9{Z}_1{Z}_5+{\mathrm{\theta}}_{10}{Z}_3{Z}_4+{\mathrm{\theta}}_{11}{Z}_3{Z}_5+{\mathrm{\theta}}_{12}{Z}_4{Z}_5\\&\quad+{\mathrm{\theta}}_{13}{Z}_1{Z}_3{Z}_4+{\mathrm{\theta}}_{14}{Z}_1{Z}_3{Z}_5+{\mathrm{\theta}}_{15}{Z}_1{Z}_4{Z}_5+{\mathrm{\theta}}_{16}{Z}_3{Z}_4{Z}_5\\&\quad+{\mathrm{\theta}}_{17}{Z}_1{Z}_3{Z}_4{Z}_5,\mathrm{SD}=1\big) \end{align*}

We inflated the coefficients for the interaction terms, approximately 4 times larger than values estimated in the VAHCS data. Under both outcome generation models, we set the coefficients for $X$ (${\mathrm{\theta}}_1$)—that is, the true value of the ACE—to 0.2. For this effect and 2000 records, the null hypothesis of no causal effect is formally rejected (*P* <.05) in approximately 80% of the simulated data sets.

#### Imposing missing data

**Figure 2:**
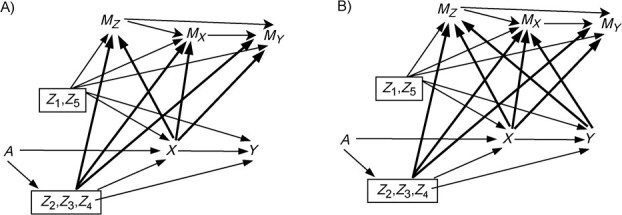
Missingness directed acyclic graphs illustrating the missingness scenarios considered in the simulation study, Victorian Adolescent Health Cohort Study, 1992-1998. The figure was adapted from Moreno-Betancur et al.^20^ For simplicity of exposition, confounders without missing data (${Z}_1$ and ${Z}_5$) are presented on a single node and confounders with missing data (${Z}_2$, ${Z}_3$, ${Z}_4$) on another single node. In addition, only 1 missingness indicator has been included for confounders with missing data (${M}_Z$), coded as 1 when any of the variables ${Z}_2$, ${Z}_3$, ${Z}_4$ have missing data and as 0 when none have missing data.

We considered missingness scenarios depicted by missingness directed acyclic graphs (m-DAGs) A and B (Figure 2).^20^^,^^29^ These were selected because they represent scenarios under which our expectations of the performance of methods were quite distinct (see Discussion section).

We imposed missingness on ${Z}_2,{Z}_3,{Z}_4,X,Y$ through generation of missingness indicators, ${M}_{Z_2},{M}_{Z_3},{M}_{Z_4},{M}_X,{M}_Y$, coded 0 if the variable was observed and 1 if it was missing. We considered variables $A,{Z}_1,{Z}_5$, which had fewer than 10% missing within VAHCS (Table 1), as fully observed in the simulation study.

For the simple scenario, the models used for generating the missingness indicators according to each m-DAG were:

$$ {M}_{Z_2}\sim \mathrm{Binomial}\left(1,{\mathrm{logit}}^{-1}\left({\mathrm{\iota}}_0+{\mathrm{\iota}}_1{Z}_1+{\mathrm{\iota}}_2{Z}_5+{\mathrm{\iota}}_3{Z}_2+{\mathrm{\iota}}_4X+{\mathrm{\iota}}_5Y\right)\right) $$

\begin{align*} {M}_{Z_3}&\sim \mathrm{Binomial}\Big(1,{\mathrm{logit}}^{-1}\big({\mathrm{\kappa}}_0+{\mathrm{\kappa}}_1{Z}_1+{\mathrm{\kappa}}_2{Z}_5+{\mathrm{\kappa}}_3{Z}_3+{\mathrm{\kappa}}_4X\\&\quad+{\mathrm{\kappa}}_5Y+{\mathrm{\kappa}}_6{M}_{Z_2}\big)\Big) \end{align*}

\begin{align*} {M}_{Z_4}&\sim \mathrm{Binomial}\Big(1,{\mathrm{logit}}^{-1}\big({\mathrm{\lambda}}_0+{\mathrm{\lambda}}_1{Z}_1+{\mathrm{\lambda}}_2{Z}_5+{\mathrm{\lambda}}_3{Z}_4+{\mathrm{\lambda}}_4X+{\mathrm{\lambda}}_5Y\\&\quad+{\mathrm{\lambda}}_6{M}_{Z_2}+{\mathrm{\lambda}}_7{M}_{Z_3}\big)\Big) \end{align*}

\begin{align*} {M}_X&\sim \mathrm{Binomial}\Big(1,{\mathrm{logit}}^{-1}\big({\mathrm{\nu}}_0+{\mathrm{\nu}}_1{Z}_1+{\mathrm{\nu}}_2{Z}_5+{\mathrm{\nu}}_3{Z}_2+{\mathrm{\nu}}_4{Z}_3+{\mathrm{\nu}}_5{Z}_4\\&\quad+{\mathrm{\nu}}_6X+{\mathrm{\nu}}_7Y+{\mathrm{\nu}}_8{M}_{Z_2}+{\mathrm{\nu}}_9{M}_{Z_3}+{\mathrm{\nu}}_{10}{M}_{Z_4}\big)\Big) \end{align*}

\begin{align*} {M}_Y&\sim \mathrm{Binomial}\Big(1,{\mathrm{logit}}^{-1}\big({\mathrm{\xi}}_0+{\mathrm{\xi}}_1{Z}_1+{\mathrm{\xi}}_2{Z}_5+{\mathrm{\xi}}_3{Z}_2+{\mathrm{\xi}}_4{Z}_3+{\mathrm{\xi}}_5{Z}_4\\&\quad+{\mathrm{\xi}}_6X+{\mathrm{\xi}}_7Y+{\mathrm{\xi}}_8{M}_{Z_2}+{\mathrm{\xi}}_9{M}_{Z_3}+{\mathrm{\xi}}_{10}{M}_{Z_4}+{\mathrm{\xi}}_{11}{M}_X\big)\Big) \end{align*}

For each missingness indicator, we set the coefficients for all confounders and the exposure to 0.9. We set the coefficient for the outcome to 0 for m-DAG A and to 0.1 for m-DAG B.

For the complex scenario, we considered 2 sets of models for generating missingness according to each m-DAG. The first set, hereafter called complex scenario 1, used the same models as for the simple scenario, detailed above. The second set, hereafter called complex scenario 2, used the following models:

\begin{align*} {M}_{Z_2}&\sim \mathrm{Binomial}\Big(1,{\mathrm{logit}}^{-1}\big({\mathrm{\iota}}_0+{\mathrm{\iota}}_1{Z}_1+{\mathrm{\iota}}_2{Z}_5+{\mathrm{\iota}}_3{Z}_2+{\mathrm{\iota}}_4X+{\mathrm{\iota}}_5Y\\&\quad+{\mathrm{\iota}}_6X{Z}_2+{\mathrm{\iota}}_7{Y}^2\big)\Big) \end{align*}

\begin{align*} {M}_{Z_3}&\sim \mathrm{Binomial}\Big(1,{\mathrm{logit}}^{-1}\big({\mathrm{\kappa}}_0+{\mathrm{\kappa}}_1{Z}_1+{\mathrm{\kappa}}_2{Z}_5+{\mathrm{\kappa}}_3{Z}_3+{\mathrm{\kappa}}_4X+{\mathrm{\kappa}}_5Y\\&\quad+{\mathrm{\kappa}}_6{M}_{Z_2}+{\mathrm{\kappa}}_7X{Z}_3+{\mathrm{\kappa}}_8{\mathrm{Y}}^2\big)\Big) \end{align*}

\begin{align*} {M}_{Z_4}&\sim \mathrm{Binomial}\Big(1,{\mathrm{logit}}^{-1}\big({\mathrm{\lambda}}_0+{\mathrm{\lambda}}_1{Z}_1+{\mathrm{\lambda}}_2{Z}_5+{\mathrm{\lambda}}_3{Z}_4+{\mathrm{\lambda}}_4X+{\mathrm{\lambda}}_5Y\\&\quad+{\mathrm{\lambda}}_6{M}_{Z_2}+{\mathrm{\lambda}}_7{M}_{Z_3}+{\mathrm{\lambda}}_8X{Z}_4+{\mathrm{\lambda}}_9{Y}^2\big)\Big) \end{align*}

\begin{align*} {M}_X&\sim \mathrm{Binomial}\Big(1,{\mathrm{logit}}^{-1}\big({\mathrm{\nu}}_0+{\mathrm{\nu}}_1{Z}_1+{\mathrm{\nu}}_2{Z}_5+{\mathrm{\nu}}_3{Z}_2+{\mathrm{\nu}}_4{Z}_3+{\mathrm{\nu}}_5{Z}_4\\&\quad+{\mathrm{\nu}}_6X+{\mathrm{\nu}}_7Y+{\mathrm{\nu}}_8{M}_{Z_2}+{\mathrm{\nu}}_9{M}_{Z_3}+{\mathrm{\nu}}_{10}{M}_{Z_4}+{\mathrm{\nu}}_{11}X{Z}_2+{\mathrm{\nu}}_{12}X{Z}_3\\&\quad+{\mathrm{\nu}}_{13}X{Z}_4+{\mathrm{\nu}}_{14}{Y}^2\big)\Big) \end{align*}

\begin{align*} {M}_Y&\sim \mathrm{Binomial}\Big(1,{\mathrm{logit}}^{-1}\big({\mathrm{\xi}}_0+{\mathrm{\xi}}_1{Z}_1+{\mathrm{\xi}}_2{Z}_5+{\mathrm{\xi}}_3{Z}_2+{\mathrm{\xi}}_4{Z}_3+{\mathrm{\xi}}_5{Z}_4\\&\quad+{\mathrm{\xi}}_6X+{\mathrm{\xi}}_7Y+{\mathrm{\xi}}_8{M}_{Z_2}+{\mathrm{\xi}}_9{M}_{Z_3}+{\mathrm{\xi}}_{10}{M}_{Z_4}+{\mathrm{\xi}}_{11}{M}_X+{\mathrm{\xi}}_{12}X{Z}_2\\&\quad+{\mathrm{\xi}}_{13}X{Z}_3+{\mathrm{\xi}}_{14}X{Z}_4\big)\Big) \end{align*}

For complex scenario 2, we set the coefficients for all confounders, the exposure, and the outcome at the same values as those used for the simple scenario, detailed above. We set the coefficients for exposure-confounder interactions ($X{Z}_2,X{Z}_3,X{Z}_4$) to 0.9, and for ${Y}^2$ to 0 for m-DAG A and 0.08 for m-DAG B.

This led to 6 scenarios overall (2 m-DAGs $\times$ 3 data-generating scenarios). The missingness proportions were the same in all missingness scenarios and approximately the same as in the real VAHCS data set, except for the outcome, which was increased to 20% (13% in VAHCS).

The overlaps between missingness proportions were adjusted so that the proportions of records excluded were 50% for CCA, 40% for Ext-TMLE+CEC, and 30% for Ext-TMLE+MCMI (Table S2).

#### Analysis of the simulated data

The target analysis aimed to estimate the ACE of $X$ on $Y$ using TMLE with data-adaptive methods adjusting for ${Z}_1,{Z}_2,{Z}_3,{Z}_4,{Z}_5$ as confounders. We used the *TMLE* package in R.^21^ We fitted the exposure and outcome models using a SuperLearner library that included a range of parametric, semiparametric, and nonparametric methods.^30^^,^^31^ The SE for TMLE was obtained using the variance of the influence function.^9^ The analysis was applied to each simulated incomplete data set alongside each of the previously described missing-data methods. For Ext-TMLE+CEC and Ext-TMLE+MCMI, we used the same SuperLearner library for the outcome missingness model. We used the *mice* package in R to implement MI.^27^ Due to computational constraints, for each MI approach, we generated 5 imputed data sets (see Discussion).^25^ Tables S3 and S4 show the variables and interaction terms included in each imputation model for MI-2-way int and MI-higher int. We used the default settings of the *mice* package for the donor pool for PMM in parametric MI approaches and for the hyperparameters for MI-CART and MI-RF.^27^

#### Evaluation criteria

We compared the performance of the approaches for handling missing data by calculating the percent relative bias, the empirical SEs, and the percent error in average model-based SE relative to the empirical SE. For all, Monte-Carlo SEs (MC-SEs) were obtained.^32^

Analyses were performed in R, version 3.6.1.^33^

## Results

### Simulation study results

#### Relative bias

**Figure 3:**
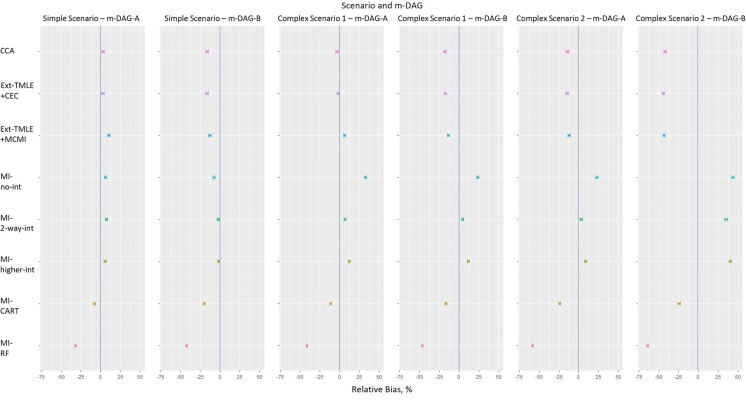
Percent relative bias (colored circles) in estimation of the average causal effect derived using different missing-data methods for simple and complex scenarios and missingness directed acyclic graphs (m-DAGs) A and B, Victorian Adolescent Health Cohort Study, 1992-1998. For all missing-data methods, targeted maximum likelihood estimation (TMLE) was implemented using SuperLearner, including the following methods: mean (the average), glm (generalized linear model), glm.Interaction (generalized linear model with 2-way interactions between all pairs of variables), bayesglm (Bayesian generalized linear model), gam (generalized additive model), glmnet (elastic net regression), earth (multivariate adaptive regression splines), rpart (recursive partitioning and regression trees), rpartPrune (recursive partitioning with pruning), and ranger (random forest). Error bars show Monte Carlo SEs. CCA, complete-case analysis (pink); Ext-TMLE+CEC, extended TMLE in a sample with complete exposure and confounders (CEC) (purple); Ext-TMLE+MCMI, extended TMLE plus the missing covariate missing indicator (MCMI) approach (blue); MI-no int, parametric multiple imputation (MI) with no interaction (predictive mean matching used to impute missing outcome) (turquoise); MI-2-way int, parametric MI with 2-way interactions (green); MI-higher int, parametric MI with 2-, 3-, and 4-way interactions (lime); MI-CART, MI using classification and regression trees (CART) (gold); MI-RF, MI using random forest (RF) (red).

In the simple scenario, under m-DAG A, CCA and Ext-TMLE+CEC yielded small biases (≤3%). Ext-TMLE+MCMI was more biased (10%) (Figure 3). These 3 approaches led to larger biases under m-DAG B (−13% to −16%). The 3 parametric MI approaches performed similarly to each other under both m-DAGs, yielding small biases (<7%). MI-CART performed similarly to the parametric MI approaches under m-DAG A (relative bias −8%), but it had larger bias under m-DAG B (−20%). Of all the approaches, MI-RF had the larger bias under both m-DAGs (−32% under m-DAG A; −42% under m-DAG B).

Biases in complex scenario 1, for both m-DAGs, were similar to those in the simple scenario, except that MI-no int had larger bias than in the simple scenario (33% under m-DAG A; 23% under m-DAG B).

In complex scenario 2, for m-DAG A, biases for non-MI methods (<|15%|), MI-CART (−24%), and MI-RF (−58%) were larger than in prior scenarios, while the 3 parametric MI approaches performed similarly to complex scenario 1. For m-DAG B, all of the non-MI and parametric MI approaches and MI-RF had larger bias than in prior scenarios (−42% to −43% for non-MI approaches, 35%-43% for parametric MI approaches, and −64% for MI-RF), while MI-CART had bias similar to that in the simple scenario and complex scenario 1.

The MC-SE for relative bias ranged from 0.81% to 1.98% across scenarios and m-DAGs.

#### Empirical SE and relative error in model-based SE

**Figure 4:**
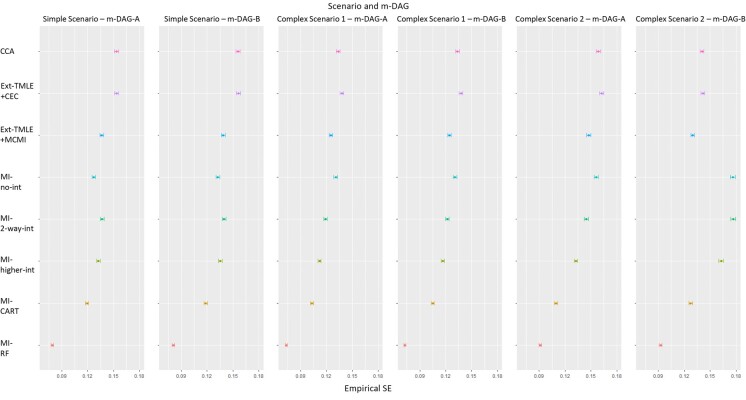
Empirical SE (colored circles) in estimation of the average causal effect derived using different missing-data methods for simple and complex scenarios and missingness directed acyclic graphs (m-DAGs) A and B, Victorian Adolescent Health Cohort Study, 1992-1998. For all missing-data methods, targeted maximum likelihood estimation (TMLE) was implemented using SuperLearner, including the following methods: mean (the average), glm (generalized linear model), glm.Interaction (generalized linear model with 2-way interactions between all pairs of variables), bayesglm (Bayesian generalized linear model), gam (generalized additive model), glmnet (elastic net regression), earth (multivariate adaptive regression splines), rpart (recursive partitioning and regression trees), rpartPrune (recursive partitioning with pruning), and ranger (random forest). Error bars show Monte Carlo SEs. CCA, complete-case analysis (pink); Ext-TMLE+CEC, extended TMLE in a sample with complete exposure and confounders (CEC) (purple); Ext-TMLE+MCMI, extended TMLE plus the missing covariate missing indicator (MCMI) approach (blue); MI-no int, parametric multiple imputation (MI) with no interaction (predictive mean matching used to impute missing outcome) (turquoise); MI-2-way int, parametric MI with 2-way interactions (green); MI-higher int, parametric MI with 2-, 3-, and 4-way interactions (lime); MI-CART, MI using classification and regression trees (CART) (gold); MI-RF, MI using random forest (RF) (red).

Across scenarios and m-DAGs, the empirical SEs using CCA and Ext-TMLE+CEC were similar (0.13-0.16) and larger than those for Ext-TMLE+MCMI (0.12-0.14) (Figure 4). The SEs obtained from the parametric MI approaches were similar to each other (0.10-0.14), to Ext-TMLE+MCMI, and MI-CART, except for complex scenario 2 under m-DAG B, where the parametric MI approaches exhibited larger SEs (0.16-0.18). The empirical SEs were similar across scenarios and m-DAGs for MI-CART (0.10-0.13) and MI-RF (0.07-0.09). MI-RF had the lowest empirical SE in all scenarios and m-DAGs (0.7-0.8). The MC-SE for empirical SEs ranged from 0.001 to 0.003.

**Figure 5:**
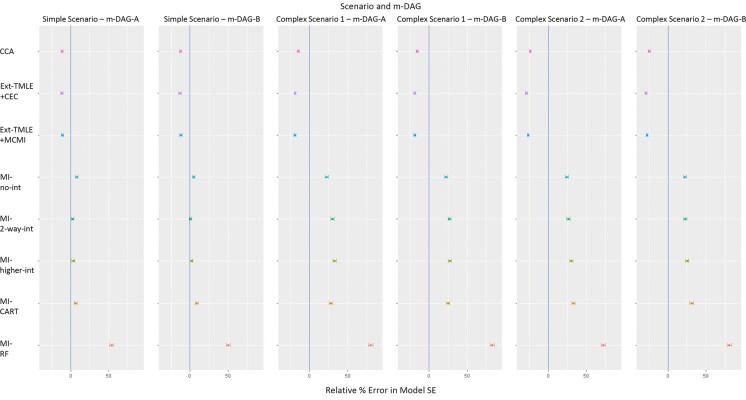
Relative percent error in model SE (colored circles) in estimation of the average causal effect derived using different missing-data methods for simple and complex scenarios and missingness directed acyclic graphs (m-DAGs) A and B, Victorian Adolescent Health Cohort Study, 1992-1998. For all missing-data methods, targeted maximum likelihood estimation (TMLE) was implemented using SuperLearner, including the following methods: mean (the average), glm (generalized linear model), glm.Interaction (generalized linear model with 2-way interactions between all pairs of variables), bayesglm (Bayesian generalized linear model), gam (generalized additive model), glmnet (elastic net regression), earth (multivariate adaptive regression splines), rpart (recursive partitioning and regression trees), rpartPrune (recursive partitioning with pruning), and ranger (random forest). Error bars show Monte Carlo SEs. CCA, complete-case analysis (pink); Ext-TMLE+CEC, extended TMLE in a sample with complete exposure and confounders (CEC) (purple); Ext-TMLE+MCMI, extended TMLE plus the missing covariate missing indicator (MCMI) approach (blue); MI-no int, parametric multiple imputation (MI) with no interaction (predictive mean matching used to impute missing outcome) (turquoise); MI-2-way int, parametric MI with 2-way interactions (green); MI-higher int, parametric MI with 2-, 3-, and 4-way interactions (lime); MI-CART, MI using classification and regression trees (CART) (gold); MI-RF, MI using random forest (RF) (red).

The model SEs were underestimated using non-MI methods and overestimated using MI methods across all scenarios and m-DAGs (Figure 5). The errors were smallest under the simple scenario and largest under complex scenario 2. Within each scenario, the performance among non-MI approaches was similar. The performance of the MI approaches was similar within each scenario, except MI-RF, which produced model SEs with considerably larger error. The MC-SE for relative percent error in model-based SEs ranged from 1.15% to 3.02%.

### Illustrative example results

We analyzed the VAHCS example using the *tmle* package in R^21^ and applied the 8 missing-data methods described. Unlike in the simulations, a small proportion of participants had missing data for parental divorce and parental education (Table 1), which were handled in the same way as missing data for the other confounders. Additionally, the auxiliary variable age had 9.3% missing data, which was multiply imputed in the MI approaches. For the MI approaches, 100 imputations were performed.

The obtained effect sizes were small, with MI-no int yielding a somewhat larger effect size and non-MI methods and MI-RF yielding smaller effect sizes (Table 2). The SEs for MI approaches were larger than those for the non-MI methods, which could be explained by the downward and upward biases in model SEs for non-MI and MI approaches, respectively, observed in our simulation study (Figure 5). For example, using the relative percent error in model SEs averaged over the 6 scenarios in the simulations, the corrected SEs in the case study would be 0.13 for CCA, 0.13 for MI-no int, and 0.11 for MI-RF.

**Table 2:** Estimated average causal effect of frequent cannabis use during adolescence on CIS-R score (standardized *z* score), derived using a TMLE approach under different missing-data methods, Victorian Adolescent Health Cohort Study, 1992-1998.

**Missing-data method** ^**a**^	**ACE (difference in mean values)** ^**b**^	**SE**	**95% CI**	**Time needed** **to run**
Complete-case	0.09	0.12	−0.14 to 0.32	16.4 s
Ext-TMLE	0.12	0.11	−0.09 to 0.33	11.2 s
Ext-TMLE+MCMI	0.13	0.13	−0.13 to 0.39	21.7 s
MI-no int	0.20	0.16	−0.11 to 0.50	4.6 min
MI-2-way int	0.16	0.17	−0.17 to 0.49	5.8 min
MI-higher int	0.18	0.16	−0.13 to 0.49	5.8 min
MI-CART	0.15	0.16	−0.16 to 0.45	11.8 min
MI-RF	0.13	0.18	−0.21 to 0.48	14.1 min

Abbreviations: ACE, average causal effect; CART, classification and regression trees; CIS-R, Clinical Interview Schedule–Revised; MCMI, missing covariate missing indicator; MI, multiple imputation; RF, random forest; TMLE, targeted maximum likelihood estimation.

^a^ Ext-TMLE, extended TMLE; Ext-TMLE+MCMI, extended TMLE plus the MCMI approach; MI no int, parametric MI with no interaction (predictive mean matching used to impute missing outcome); MI-2-way int, parametric MI with 2-way interactions; MI-higher int, parametric MI with 2-, 3-, and 4-way interactions; MI-CART, MI using CART; MI-RF, MI using RF.

^b^ The ACE was estimated as the difference in the mean potential outcome under exposure and under no exposure.

In the VAHCS example, the outcome (mental health in young adulthood) might well have influenced its own missingness, in which case neither of the considered m-DAGs in the simulation study are plausible for our example and we would expect all of the considered missing-data methods to be biased.^20^

## Discussion

We compared methods for handling missing data when estimating the ACE using TMLE with data-adaptive approaches. We considered 6 scenarios with different exposure and outcome generation models (presence/absence of confounder-confounder interaction terms) and missingness mechanisms (whether the outcome influenced missingness in other variables and presence/absence of interaction/nonlinear terms in missingness models). CCA and Ext-TMLE+CEC had small bias under m-DAG A (where the outcome did not influence missingness in other variables), and large bias otherwise. MI-no int—the default model in most MI software—had large bias in complex scenarios 1 and 2 (when the exposure/outcome generation models included interactions) and small bias otherwise (regardless of m-DAG). MI-2-way int and MI-higher int performed best in terms of bias and variance across all settings, except for m-DAG B in complex scenario 2 (where a nonlinear outcome term influenced missingness in other variables). MI-RF had consistently large bias across the 6 scenarios. MI-CART had small bias under m-DAG A in the simple scenario and complex scenario 1, and large bias otherwise.

Based on previous investigations in a setting without effect modification, we determined that for m-DAG A, where the outcome did not influence missingness in any variable, the ACE was identifiable (or “recoverable”).^20^ Further, because auxiliary variables did not influence missingness in any variable, we expected both CCA and an appropriate implementation of MI to yield low bias.^34^ Indeed, CCA (and Ext-TMLE+CEC) produced estimates with small bias for m-DAG A across all data-generation scenarios. In addition, implementations of parametric MI that were approximately compatible with the analysis method (ie, all parametric MI procedures in the simple scenario, and MI including interaction terms in complex scenarios) returned estimates with little bias, while an inappropriate MI method (eg, MI-no int in our complex scenarios) was considerably more biased.

Contrary to CCA and Ext-TMLE+CEC, the Ext-TMLE+MCMI approach had higher bias under m-DAG A. A key assumption under which the MCMI approach has been shown to be unbiased is when the exposure or outcome only depends on the confounder when the confounder is observed.^23^^,^^24^ We did not consider missingness scenarios where this held, because this assumption is implausible in a prospective cohort study, like VAHCS, where the data are not used for medical decision-making.

For m-DAG B, the ACE was determined to be nonrecoverable, but since the outcome did not influence its own missingness, based on a previous simulation study,^20^ we speculated that an implementation of MI that was tailored to the analysis method may offer some bias reduction in comparison with CCA. We observed that parametric MI approaches including interactions performed better than all other approaches in terms of bias for the simple scenario and complex scenario 1. For complex scenario 2, where the missingness models included exposure-confounder interactions and a quadratic term for the outcome, all parametric MI approaches were highly biased. MI-CART outperformed parametric MI approaches in this scenario, but it was still moderately biased.

Within the FCS framework, recursive partitioning techniques, such as CART and RF, have been suggested as alternative approaches that could automatically incorporate interactions and nonlinearities in the imputation process.^28^ Previous simulation studies have shown that MI using CART performs better than parametric MI without interaction terms.^28^^,^^35^ However, in these studies, the target analysis was a correctly specified outcome regression model with interactions, and biases in estimates of the main effects were not that different following MI using CART or parametric MI without interaction. These studies imposed missingness in the outcome only^28^ or outcome and covariates.^35^ In both, missingness depended on fully observed variables. In the present study, the only setting where MI-CART outperformed parametric MI approaches was for m-DAG B in complex scenario 2. In all scenarios, bias in the ACE estimates following MI-RF was larger than MI-CART, consistent with Doove et al’s results.^28^ We speculate that this might have been because in the implementation of MI-RF, we used a small number of randomly preselected predictor variables to split the sample at each node, which might have negatively affected prediction accuracy.^36^ Specifically, the *ranger* package used within R’s *mice* package for implementing RF uses, as the default number of preselected variables to split at each node, the square root of the total number of variables in the imputation model, which was 2 in our simulation.^28^ CART, on the other hand, does not involve variable preselection.^36^

In this study, non-MI approaches underestimated the model SE, which was not surprising. If both the exposure and outcome models are correctly specified and the Donsker class condition is satisfied, TMLE is an asymptotically linear estimator and its variance can be obtained based on the variance of the influence curve.^9^^,^^10^ It is unclear, however, whether the Donsker class condition is met when data-adaptive approaches are used for the exposure and outcome models.^37^ This leads to bias in variance estimation, as has been observed here and in other simulation studies.^31^^,^^37^^,^^38^ It is an ongoing area of research to develop approaches to tackle it, such as cross-validated TMLE, which allows asymptotic linearity to be established without the Donsker class condition.^10^ Additionally, Rubin’s MI variance estimator is expected to perform poorly in the presence of incompatibility,^39^ which might explain the overestimation of model SEs for the MI approaches. Incompatibility is the key challenge for using MI with TMLE with data-adaptive approaches, in terms of bias of point estimates as discussed previously, but even more so for bias in variance estimates. A promising alternative approach for obtaining SEs for MI in the presence of incompatibility was recently proposed using the bootstrap,^39^ but we did not explore this because of computational constraints. Incompatibility between the models used in MI and TMLE may also compromise the double-robustness property of TMLE.^40^ Other approaches, such as the Ext-TMLE approach considered in this paper to handle missing outcome data, and an alternative likelihood parameterization to construct doubly robust estimators in adjusting for confounding and missing data in the presence of missing confounder data,^40^ guarantee double robustness under extended assumptions.

Our simulation study was broadly based on VAHCS. We evaluated the performance of missing-data methods under various missingness mechanisms. To describe what variables influenced missingness, we used m-DAGs because the standard classification of data being missing completely at random, missing at random (MAR), or missing not at random is difficult to comprehend and substantively assess when there is missingness in multiple variables. In addition, although it is possible to estimate key parameters unbiasedly if the MAR assumption holds, MAR is not necessary for unbiased estimation.^20^^,^^34^ We did not consider missingness mechanisms where the outcome influenced its own missingness, under which none of the approaches could be expected to perform well. For each MI approach, due to computational constraints we generated 5 completed data sets in the simulation study, which is fewer than we would do in practice.^25^ We do not expect this to have affected the comparison between MI approaches, but it could have affected comparison of non-MI methods with MI methods. Our simulated data had a relatively simple structure across the assessed scenarios. Extensions of our study could investigate the performance of these missing-data methods for data sets with high-dimensional confounders, binary outcomes, and more complex m-DAGs including longitudinal auxiliary variables.

## Conclusion

We evaluated the performance of 8 available approaches to handling missing data when estimating the ACE using TMLE with data-adaptive approaches under various data-generation scenarios and missingness mechanisms. Our results highlight the importance of considering the missingness mechanism and compatibility with the analysis method when choosing a method for handling missing data. In many settings, a parametric MI approach that incorporates interactions and nonlinearities is expected to perform well in the context of TMLE with data-adaptive approaches.

## Supplementary Material

Web_Material_kwae012

## Data Availability

Data from VAHCS are not publicly available. Persons interested in replicating these findings are welcome to contact the corresponding author (S.G.D.) or the VAHCS study team (https://www.mcri.edu.au/research/projects/2000-stories/information-researchers). Simulation study code can be made available upon request to the corresponding author.
