# Supplementary material for: Handling missing data when estimating causal effects with targeted maximum likelihood estimation
Source: Am J Epidemiol. 2024 Feb 22;193(7):1019–30. doi: 10.1093/aje/kwae012 (PMC11228874; doi:10.1093/aje/kwae012)
Supplement: Web_Material_kwae012 [file web_material_kwae012.pdf]

Supplementary Data

"Handling missing data when estimating causal effects with targeted maximum likelihood estimation"

S. Ghazaleh Dashti, Katherine J. Lee, Julie A. Simpson, Ian R. White, John B. Carlin, Margarita Moreno-Betancur

The contents of this document are as follows:
Table S1 Coefficient values used to simulate the variables and missingness indictors under
Table S2 Description of variables in the simulated data (averaged over 2000 simulations)
Table S3 The variables and interaction terms included in each imputation model for a multiple imputation approach that included all two-way interactions
Table S4 The variables and interaction terms included in each imputation model for a multiple imputation approach that included all two-, three-, and four-way interactions

Table S1 - Coefficient values used to simulate the variables and missingness indictors under

	Model for	Regression coefficent of<br>Intercept	Z1	Z2	Z3	Z4	Z5	X	Y	A	MZ2**	MZ3**	MZ4**	MX**
Complete data	*Z1*	-1.30												
	Z2	-1.90								0.40				
	Z3	0.40								0.70*				
	Z4	-0.60								'-0.70*				
	Z5	-0.50												
							**Simple scenario**							
	X	-2.9*	1.30	1.90	0.40	0.20*	-0.30			0.70*				
	Y	-0.7*	0.10	0.40*	0.70	0.20*	0.30*	0.20*						
							**Complex scenario 1 and 2**							
	X	`-2.4*	1.30	1.90	0.40	0.20	-0.30							
	Y	-0.70*	0.10	0.40*	0.70	0.20*	0.30*	0.20*						
							**Simple and complex scenario 1 and 2**							
DAG A	*M Z2*	-1.75	0.90	0.90			0.90	0.90						
	MZ3	-5.70	0.90		0.90		0.90	0.90			4.10			
	MZ4	-4.80	0.90			0.90	0.90	0.90			3.20	2.00		
	MX	-3.80	0.90	0.90	0.90	0.90	0.90	0.90			1.50	1.50	1.50	
	MY	-3.20	0.90	0.90	0.90	0.90	0.90	0.90			-0.60	0.10	0.10	0.10
DAG B	*M Z2*	-1.75	0.90	0.90			0.90	0.90	0.10					
	MZ3	-5.95	0.90		0.90		0.90	0.90	0.10		4.30			
	MZ4	-4.80	0.90			0.90	0.90	0.90	0.10		3.50	1.30		
	MX	-3.80	0.90	0.90	0.90	0.90	0.90	0.90	0.10		1.50	1.50	1.50	
	MY	-3.20	0.90	0.90	0.90	0.90	0.90	0.90			-0.60	0.10	0.10	0.20

*** For the complex scenarios 1 and 2 models for X and Y also included interactions as follows:**

	z1z3	*z1z4*	*z1z5*	*z3z4*	*z3z5*	*z4z5*	*z1z3z4*	*z1z3z5*	*z1z4z5*	*z3z4z5*	*z1z3z4z5*
					Complex scenario 1 and 2						
X	-3.20	-2.30	-1.00	-1.20	0.50	-2.90					
Y	-0.90	2.00	0.10	0.20	0.70	-0.20	-2.40	-2.00	-0.30	-0.80	3.40

*** For the complex scenario 2 models for missingness indicators included interaction and non-linear terms as follows:**

		xz2	xz3	xz4	y^2
					Complex scenario 2
DAG A	*M Z2*	0.90			
	MZ3		0.90		
	MZ4			0.90	
	MX	0.90	0.90	0.90	
	MY	0.90	0.90	0.90	
DAG B	*M Z2*	0.90			0.08
	MZ3		0.90		0.08
	MZ4			0.90	0.08
	MX	0.90	0.90	0.90	0.08
	MY	0.90	0.90	0.90	

* These parameter values were modified from what is in the VAHCS data as follows: In generating confounders *Z3* and *Z4* , we changed the coefficient value for *A* from what it was in VAHCS, so that it was a stronger auxiliary variable, and modified the intercepts so that the prevalence of the variables remained the same as in the VAHCS dataset. For the exposure model, we modified the coefficient value for *A* , so that it was a stronger auxiliary variable, and the coefficient value for *Z4* , so that it was less strongly associated with *X* . We modified the intercept so that the prevalence of *X* was approximately 15% in the simulated data (12% in VAHCS) in all scenarios. For the outcome model, we modified the coefficient values for *Z2* , *Z4* , and *Z5* , so that they were stronger confounders (the coefficient values for the confounders ranged from 0.1 (for *Z1*) to 0.7 (for *Z3*) in the simulation study). Under all outcome generation models, we set the coefficient value for *X* (θ1), which is the true value of the ACE, to 0.2. We modified the intercept in the outcome model so that the mean of *Y* remained 0.

**As shown in the models provided in the mauscript, the regression model for generating *M(Z3)* included the missingness indicator *M(Z2*), the model for *M(Z4*) included *M(Z2)* and *M(Z3)* , the model for *M(X)* included *M(Z2)* , *M(Z3)* , and *M(Z4)* , and the model for *M(Y)* included all the preceding missingness indicators. We modified the coefficient values for these missingness indicators and the intercepts so that the missingness proportion for each variable and the overall proportions with missing data were the same across all missingness scenarios.

Table S2– Description of variables in the simulated data (averaged over 2000 simulations)

	Variable	Type	Notation	%* coded 1 or mean (SD)	% with missing data
Confounder	Parental divorce	Binary	*Z1*	21	0
	Antisocial behaviour	Binary	*Z2*	14	30
	Depression and anxiety	Binary	*Z3*	59	15
	Alcohol use	Binary	*Z4*	37	20
	Parental education	Binary	*Z5*	38	0
Exposure	Frequent cannabis use	Binary	*X*	15	30
Outcome	CIS-R total score	Continuous	*Y*	0 (1)	20
With missing exposure or confounder data					40
With any missing data					50

Abbreviations SD standard deviation; CIS-R revised clinical interview schedule *Proportions reported in the simulated complete data

Table S3 - The variables and interaction terms included in each imputation model for a multiple imputation approach that included all two-way interactions

Variable imputed	Variables included in imputation model																					
	A	*Z1*	*Z2*	*Z3*	*Z4*	*Z5*	*X*	*Y*	*XY*	*XZ1*	*YZ1*	*XZ3*	*YZ3*	*XZ4*	*YZ4*	*XZ5*	*YZ5*	*Z1Z3*	*Z1Z4*	*Z1Z5*	*Z3Z4*	*Z3Z5*	*Z4Z5*
Z2	1	1	0	1	1	1	1	1	1	1	1	1	1	1	1	1	1	1	1	1	1	1	1
Z3	1	1	1	0	1	1	1	1	1	1	1	0	0	1	1	1	1	0	1	1	0	0	1
Z4	1	1	1	1	0	1	1	1	1	1	1	1	1	0	0	1	1	1	0	1	0	1	0
X	1	1	1	1	1	1	0	1	0	0	1	0	1	0	1	0	1	1	1	1	1	1	1
Y	1	1	1	1	1	1	1	0	0	1	0	1	0	1	0	1	0	1	1	1	1	1	1

Table S4 - The variables and interaction terms included in each imputation model for a multiple imputation approach that included all two-, three-, and four-way interactions

Variable imputed	Variables included in imputation model																											
	A	*Z1*	*Z2*	*Z3*	*Z4*	*Z5*	*X*	*Y*	*XY*	*XZ1*	*YZ1*	*XZ3*	*YZ3*	*XZ4*	*YZ4*	*XZ5*	*YZ5*	*Z1Z3*	*Z1Z4*	*Z1Z5*	*Z3Z4*	*Z3Z5*	*Z4Z5*	*Z1Z3Z4*	*Z1Z3Z5*	*Z1Z4Z5*	*Z3Z4Z5*	*Z1Z3Z4Z5*
Z2	1	1	0	1	1	1	1	1	1	1	1	1	1	1	1	1	1	1	1	1	1	1	1	1	1	1	1	1
Z3	1	1	1	0	1	1	1	1	1	1	1	0	0	1	1	1	1	0	1	1	0	0	1	0	0	1	0	0
Z4	1	1	1	1	0	1	1	1	1	1	1	1	1	0	0	1	1	1	0	1	0	1	0	0	1	0	0	0
X	1	1	1	1	1	1	0	1	0	0	1	0	1	0	1	0	1	1	1	1	1	1	1	1	1	1	1	1
Y	1	1	1	1	1	1	1	0	0	1	0	1	0	1	0	1	0	1	1	1	1	1	1	1	1	1	1	1
